# Antibiotic Prescribing in Primary Care and Antimicrobial Resistance in Patients Admitted to Hospital with Urinary Tract Infection: A Controlled Observational Pilot Study

**DOI:** 10.3390/antibiotics3010029

**Published:** 2014-01-22

**Authors:** Ceire Costelloe, O. Martin Williams, Alan A. Montgomery, Colin Dayan, Alastair D. Hay

**Affiliations:** 1 Centre for Primary Care and Public health, Blizzard Institute, Queen Mary University of London, London E1 2AB, UK; 2 Health Protection Agency Microbiology Services, United Hospital Bristol Trust, Bristol Royal Infirmary, Marlborough Street, Bristol BS1 3NU, UK; E-Mail: martinx.williams@uhbristol.nhs.uk; 3 Faculty of Medicine and Clinical Sciences, University of Nottingham, Nottingham NG7 2UH, UK; E-Mail: alan.montgomery@nottingham.ac.uk; 4 Institute of Molecular & Experimental Medicine, Cardiff University School of Medicine, Wales Heart Research Institute, Heath Park, Cardiff CF14 4XN, UK; E-Mail: dayancm@cf.ac.uk; 5 Centre for Academic Primary Care, NIHR School for Primary Care Research, School of Social and Community Medicine, University of Bristol, Canynge Hall, Bristol BS8 2PS, UK; E-Mail: alastair.hay@bristol.ac.uk

**Keywords:** antibiotics, primary care, antimicrobial resistance, urinary tract infection

## Abstract

There is growing evidence that primary care prescribed antibiotics lead to antibiotic resistance in bacteria causing minor infections or being carried by asymptomatic adults, but little research to date has investigated links between primary care prescribed antibiotics and resistance among more serious infections requiring hospital care. Knowledge of these effects is likely to have a major influence on public expectations for, and primary care use of, antibiotics. This study aimed to assess the feasibility of recruiting symptomatic adult patients admitted to hospital with urinary infections and to link primary and secondary data information to investigate the relationship between primary care prescribed antibiotics and antimicrobial resistance in these patients. A microbiology database search of in patients who had submitted a urine sample identified 740 patients who were potentially eligible to take part in the study. Of these, 262 patients did not meet the eligibility criteria, mainly due to use of a urinary catheter (40%). Two-hundred and forty three patients could not be recruited as the nurse was unable to visit the patients prior to discharge, as they were too unwell. Eighty patients provided complete information. Results indicate that there is evidence that prior antibiotic use is associated with resistant infections in hospital patients. A fully powered study, conducted using routinely collected data is proposed to fully clarify the precision of the association.

## 1. Introduction

Resistance to antibiotics is a major threat to public health, and in the European Union, about 25,000 patients die in hospital each year from infections caused by selected multidrug-resistant bacteria and the associated costs are estimated at about 1.5 billion Euros per year [1]. Of significant concern, is the rate at which bacteria are becoming resistant, which is outstripping the rate at which new antibiotics are being developed [2]. General Practitioners (GPs) are responsible for 80% of all antibiotics prescribed to humans [3]. Patient expectations for antibiotics are a powerful determinant of prescribing [4] and for some GPs and patients, antibiotic resistance is seen only as a theoretical [4] or minimal [5] risk. The relationship between primary care antibiotic prescribing and resistance is complex and incompletely understood. Previous studies have established the relationship between the use of antibiotics in primary care and the asymptomatic carriage of resistant bacteria in adults [6,7,8] and children [9]. A more recent systematic review [10] found 18 studies showing consistent, temporal relationships between prescribing and resistance in bacteria causing relatively minor infections and in asymptomatic subjects. These studies contribute important observations to the existing literature that improve our understanding of how antibiotic resistance develops [11], they may or may not be sufficient to persuade clinicians [12] or patients [13] of the value of judicious antibiotic use in relation to reducing antibiotic resistance among patients with symptomatic infections, especially those requiring secondary care treatment.

Therefore, this paper reports a pilot and feasibility study, which aimed to investigate the relationship between primary care prescribed antibiotics and antimicrobial resistance, among symptomatic adult patients admitted to hospital with infection. Knowledge of these effects, and their associated economic impact, is likely to have a major influence on public expectations for, and primary care use of, antibiotics.

The recently published European Strategic Action Plan on Antimicrobial Resistance called for research using links between primary and secondary care to reduce morbidity and mortality associated with antimicrobial resistance, to promote prudent use of antibiotics and to raise awareness of the emergence and spread of antimicrobial resistance across all healthcare settings [14]. The study aimed to use routinely collected data stored within the patient primary and secondary care record, and a key component of the study was to examine the feasibility of linking this information at the individual patient level. Specifically our objectives were to: determine optimal recruitment, consent and data collection methods; describe the prevalence of antimicrobial susceptibility/resistance among isolated *Escherichia coli* (*E. coli*) from patients with urinary tract infection (UTI); and to examine primary care antibiotic prescribed within 12 months of admission and resistance to inform the sample size calculation of future studies.

## 2. Results and Discussion

**Figure 1:**
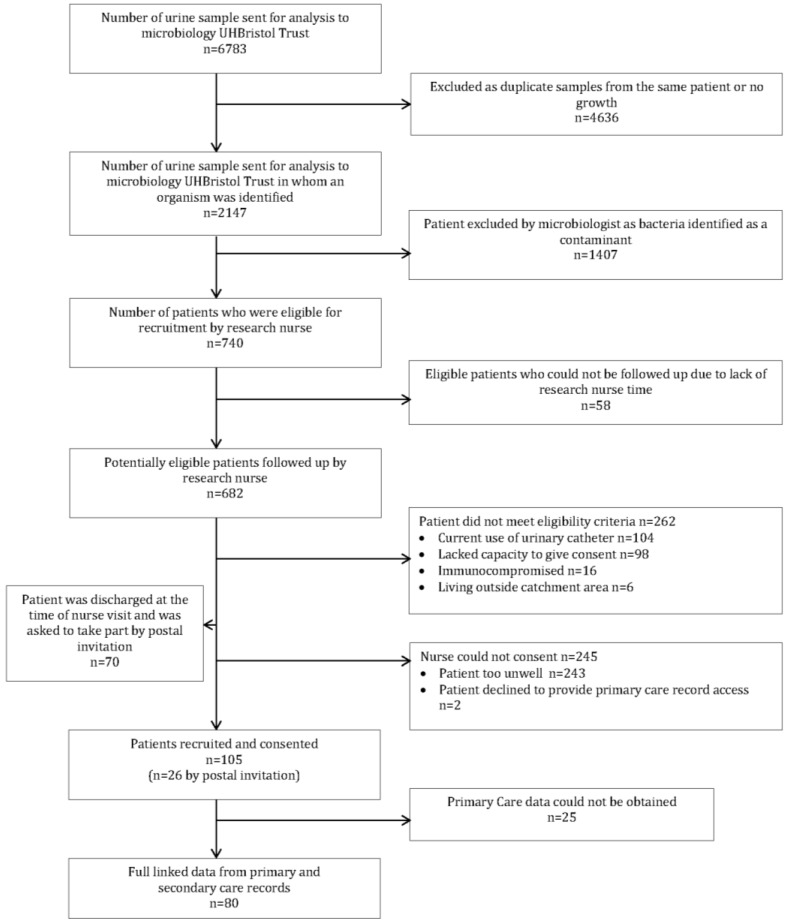
Diagram showing flow of participants through the study.

Between October 2010 and July 2011, 6,783 urine samples were submitted for microbiology testing at UHBristol Trust laboratory. Of these, 2,147 grew an organism, of which 1,407 (65%) were ineligible (contaminant). The remaining 740 samples were from patients who were then potentially available to be screened for study eligibility (see Figure 1). Thirty-three female patients and twenty-five male patients could not be followed up due to lack of nurse time (n = 58). Thirty-eight percent of eligible patients were subsequently identified as ineligible and a further 36% could not meet with the research nurse as they were too unwell. Complete primary care data were available for 80 participants.

### 2.1. Practices and Participants

Patients were registered with a total of 25 GP practices with the majority of practices contributing one patient, and six practices having more than three patients recruited to the study. Mean (SD) participant age was 63.4 (20.1), with patient age ranging from 20–92 years. Sixty-four percent of the study cohort was female and 73% had been administered antibiotics in secondary care. The majority of these (86%) were given penicillin and/or trimethoprim (82%). Other characteristics of the study population are detailed in Table 1.

**Table 1:** Characteristics of patient participating in study.

Variable	n(%) n = 105
Age (mean(SD))	63.4 (20.1)
Age category	-
20–49	24(23)
50–74	38(36)
75–84	25(24)
85+	18(17)
Female	67(64)
Secondary care antibiotic use	77(73)
Trimethoprim ^1^	63(82)
Co-amoxiclav ^1^	22(29)
Amoxicillin ^1^	66(86)
Nitrofurantoin ^1^	3(4)
Visited hospital in previous 12 months	53(50)
Antibiotic use in previous 12 months	-
Yes	21(20)
Unknown ^2^	79(75)
Co-morbidity	-
Asthma	14(13)
Diabetes mellitus	16(15)
COPD	3(3)
History of smoking	46(44)

^1^ Denominator = 77; ^2^ patient could not recall.

Table 2 details the microbiology results for 105 recruited patients. *E. coli* was the most common organism. Thirty-three and 24 percent of urinary isolates tested were resistant to trimethoprim and amoxicillin respectively.

Primary care data on antibiotic use was obtained for 63% (n = 80) of the recruited sample. Table 3 details the number of antibiotic used by patients in the previous 12 months. Thirty-seven percent of participants had no antibiotic prescriptions in the previous 12 months. Twenty-three percent of participants had three or more course of antibiotics prescribed in the previous 12 months.

Unadjusted regression analysis showed that prescription of an antibiotic course in the previous 12 months was associated with presence of a urinary isolate resistant to trimethoprim (OR 3.58 95% CI 1.18 to 10.9, Table 4).

**Table 2:** Organisms identified in patient urine samples.

**Organism identified**	**n (%), n = 105**
*Escherichia coli*	75 (71)
*Proteus* spp.	10 (10)
*Coliform*	20 (19)
**Resistance (all urinary isolates)**	**n = 105**
Trimethoprim	35 (33)
Amoxicillin	25 (24)
Ciprofloxacin	10 (10)

**Table 3:** Primary care antibiotic courses in the previous 12 months.

**Antibiotic type**	**n (%) n = 154 ^a^**
Amoxicillin	20 (13)
Co-amoxiclav	16 (10)
Flucloxacillin	13 (8)
Nitrofurantoin	28 (18)
Trimethoprim	31 (20)
Ciprofloxacin	17 (11)
Erythromycin	15 (10)
Clarithromycin	4 (3)
Other	10 (7)
**Number of courses**	**n = 80**
0	31 (39)
1	22 (27)
2	6 (8)
3–5	12 (15)
6+	9 (11)

^a^ Number of courses prescribed n = 154 for n = 80 patients. Twenty-seven patients had more than one course of antibiotic prescribed in the previous 12 months.

**Table 4:** Crude association between prescription of any antibiotic in the previous 12 months and trimethoprim resistance in patients admitted to hospital with suspected infection.

Antibiotic prescribed n = 80	Resistant n = 25 (31%)	Susceptible n = 55 (69%)	Crude OR 95% CI
Yes 49 (61)	20 (80%)	29 (53%)	3.58
No 31 (39)	5 (20%)	26 (47%)	1.18 to 10.9

### 2.2. Discussion

This study has demonstrated that recruitment of patients in secondary care is feasible, but requires significant staff resources. Data linkage methods, once patient consent had been obtained, was a successful method of carrying out this research. Primary Care data was obtained from the GP practices and was facilitated by local Primary Care Research network support. Using “opt-in” consent methods led to a high proportion (36%) of potentially eligible patients not being given the opportunity to participate in the study, and incomplete data where patients provided insufficient information on their GP practice. This has negative outcomes not only for the generalizability of the study findings but, given that many of these patients were also too unwell to give consent, excludes patients suffering from more severe resistant infections. Furthermore, results of the pilot study showed that most patients were supportive of the research, with just two (0.3%) potentially eligible participants declined to take part in the research, as they were concerned about giving access to their medical records. This suggests that the opt-in consent method may not be the most scientifically robust or efficient method. Given that most of the data used in this study are routinely collected in primary and secondary care, with appropriate data security safeguards and regulatory approvals, this research could be conducted without any direct patient involvement.

Results from a univariate analysis suggest that the primary care prescription of antibiotic in the 12 months prior to admission was associated with the presence of trimethoprim resistant isolates in patients admitted to hospital with UTI. This association appears clinically important, but needs to be adjusted for potential confounders (such as recent hospital admission) in future, more powerful, studies.

In addition, in any future study sample size should be sufficient to allow for comparison between resistance to specific antibiotics. An important factor, which was identified in this study, is that resistance testing is not standardized within the secondary care setting. A further limitation of this study is that the primary reason for admission was unknown, reflected in the variety of antibiotic therapies recorded. This information should be included in a future study. A large proportion of the study sample was excluded as they had a urinary catheter, which threatens the generalizability of study findings. In a future fully powered study, the sample size should allow for subgroup analysis—for example, investigating the association between primary care antibiotic and resistance in patients who have a catheter or patients who have co-morbidities.

Our pilot study produced results similar to those seen elsewhere. One Spanish study examined susceptibility to a number of antibiotics in patients treated by emergency services, who had been diagnosed with community acquired UTI. Patients who had been prescribed nalidixic acid or fluoroquinolones within the previous three months were found to have significantly higher resistance rates than those who had no previous primary care antibiotics [15]. A second study found that the odds of *E. coli* resistance to co-amoxiclav were four times higher comparing French patients hospitalized for urinary tract infection with and without co-amoxiclav exposure in the month prior to admission [16]. Finally, a cohort study retrospectively studied 24 adults with bacteraemic pneumonia (25 episodes) due to penicillin-resistant pneumococci compared with 48 patients with bacteraemic pneumonia caused by penicillin-sensitive pneumococci. The 24 patients with penicillin-resistant pneumococci had a significantly higher: incidence beta-lactam antibiotic use during the previous three months, hospitalization during the previous three months, nosocomial pneumonia and episodes of pneumonia during the previous year [17]. However, these studies are all relatively small and resistance rates vary substantially across Europe with Spain having higher rates than typically seen in the UK.

### 2.3. Implications for Research and Clinical Practice

Since much of the study data collected could have been collected from the microbiology database and the patient’s primary and secondary care records, database linkage methods might be more efficient for future studies. This would negate the need to recruit acutely unwell patients in the secondary care setting. Results from the study suggest that the decision to prescribe antibiotics in primary care can influence antibiotic resistance status in patients when they are admitted to secondary care, but needs to be confirmed in a fully adjusted, powered study. Further knowledge of association between primary care antibiotic prescribing and the development of resistant infections at an individual level will better inform shared GP-patient decision making in fully weighing up the pros and cons of prescribing and using antibiotics. This will benefit patients by discouraging the inappropriate prescription of antibiotics and help encourage the virtuous cycle of reduced antibiotic use and reduced bacterial resistance.

## 3. Experimental

### 3.1. Design, Setting and Participants

The study was a controlled observational pilot study including adult patients being treated for a microbiologically confirmed urinary tract infection (UTI) at the University Hospital Bristol Trust.

### 3.2. Identification of Bacteria

The microbiology database was searched daily for samples submitted within 48 h of admission in which a bacterium was isolated. To be included urines had to have both: a significant positive culture and either a clinical descriptor for a urinary tract infection (cystitis or pyelonephritis) or positive ward-based urine dip stick result. In the laboratory, a positive leukocyte esterase, and a significant growth (104–105 or >105 cfu/mL) of a single or two organisms would be considered significant. Three or more organisms were regarded as contaminants and were excluded.

### 3.3. Microbiology Methods

Bacterial identification was based on appearance in chromogenic agar or by using the automated VITEK II system. Standard British Society for Antimicrobial Chemotherapy (BSAC) [18] disc testing methods were used to determine antibiotic resistance status for any bacterium isolated from patient sample within the UHBristol Trust laboratories. Disc testing methods were used to detect resistance to a variety of antibiotics. As we used data produced from standard NHS procedures not all samples were tested for resistance to the same antibiotics.

### 3.4. Patient Recruitment and Consent

The hospital’s microbiology database was searched to ascertain the location (which hospital ward or discharged) of patients in whom bacteria had been identified. The Research Nurse located each potentially eligible patient and asked permission from the patients’ clinical team to approach the patient to provide information about the study. Patients were asked to give consent for their primary and secondary care medical records to be used to extract study data. In addition, patients were asked to complete a brief baseline questionnaire giving details of: hospital visits in the previous 12 months; antibiotic use in the previous 12 months; co-morbidities; current medication and socio-demographic information. If patients had been discharged before the nurse had an opportunity to recruit then an invitation letter and study material was sent from the microbiology team to the patient by post. Patients were ineligible if they had previously taken part in the study or had insufficient capacity to give informed consent, or if the sample had been obtained from a urinary catheter. If patients were: under 18 years of age; immunocompromised or unable to read and understand English; they were also excluded Ethical approval was given to search the primary care notes of patients from Bristol, North Somerset or South Gloucester Primary Care Trusts regions. Therefore, patients who were outside that catchment area were excluded.

### 3.5. Primary Care Antibiotic Data

GPs were informed by letter of their patient’s participation in the study. GPs were asked to complete a proforma to collect computer recorded primary care antibiotic prescribing and consultations in the 12 months prior to admission and six weeks post hospital discharge. In particular, the type, the number of units (capsules, tablets or suspension volume) per dose, the strength per unit, the total number of units and the dosing instructions was be collected for all courses of prescribed systemic antibiotics.

### 3.6. Outcome Measures

The main outcome of the study was to determine optimal recruitment, consent and data collection methods. In addition, we aimed to describe the prevalence of antimicrobial susceptibility/resistance among bacteria isolated from urine samples.

Summary statistics were used to describe recruitment and data attrition rates and demographics, antibiotic exposure and outcomes of individuals with susceptible and resistant bacteria. A separate binary variable was created to represent if an organism showed resistance to trimethoprim (Yes/No). Prior exposure to antibiotics in the 12 months before admission was recorded as a binary variable (Yes/No).

The pilot study was designed to estimate crude strengths of association to inform the sample size calculation of a future, definitive study. A logistic regression model was used to estimate the unadjusted strength of association between prior primary care antibiotic exposure and antibiotic resistance in the study and measures of variability between antibiotic exposure and resistance [19].

## 4. Conclusions

Our study has demonstrated an important possible relationship between primary care prescribed antibiotics and trimethoprim resistance in patients admitted to secondary care with UTI. This should be replicated in a fully powered study. We have also demonstrated that the recruitment methods are feasible, but that the study could be more efficiently conducted using routinely collected data. These data could be easily available for research purposes, enabling ongoing monitoring of resistant infections in hospitals. Improvements in database connections between healthcare settings could enable this information to be fed between primary care and secondary care clinicians—thereby leading to more appropriate antibiotic use both within primary and secondary health care settings.
